# Influence of subcutaneous implantable defibrillators on cardiovascular magnetic resonance image quality in pediatric patients

**DOI:** 10.1016/j.hrcr.2022.04.014

**Published:** 2022-04-26

**Authors:** Sharib Gaffar, Anthony C. McCanta, Pierangelo Renella

**Affiliations:** ∗ Pediatrics Residency Program, CHOC Children’s Hospital, Orange, California; † Department of Pediatrics, University of California Irvine School of Medicine, Orange, California; ‡ Division of Cardiology, CHOC Children’s Hospital, Orange, California; § Department of Radiological Sciences, Ronald Reagan–University of California Los Angeles Medical Center, Los Angeles, California

**Keywords:** Subcutaneous implantable cardioverter-defibrillator, ICD, Cardiac magnetic resonance imaging, Arrhythmia, Cardiomyopathy, Congenital heart disease, Image quality

**Key Teaching Points**

• Subcutaneous implantable cardioverter-defibrillators (SICDs) can hamper the utility of cardiac magnetic resonance imaging (CMR) owing to image artifact they can produce. Depending on the clinical question, patient and diagnosis-specific imaging protocols may be feasible.
• For those patients requiring evaluation of cardiac function and myocardial scarring, it may be prudent to undergo CMR prior to implantation of an SICD, or more generally, prior to implantation of any cardiovascular implantable electronic devices.
• In pediatric patients with SICDs, significant distortion and artifact were notable over the left ventricle, preventing the CMR from answering the clinical questions for which it was ordered. The right ventricle was relatively spared in terms of visualization—in this scenario, CMR may have utility if the clinical questions are more directed toward right ventricular function or right ventricular myocardial scarring.
• The imaging examples provided offer a glimpse into the type and extent of distortion an SICD can impose upon CMR images and may change the pediatric cardiologists’ clinical practice in terms of timing or indication for ordering CMR in these patients.
• There were no adverse outcomes or device malfunctions immediately post CMR in the 5 cases with SICDs.

## Introduction

Pediatric and adult patients with ischemic and nonischemic cardiomyopathy commonly require implantable cardioverter-defibrillators (ICDs) for arrhythmia management.^1^ Cardiac magnetic resonance imaging (CMR) is an invaluable diagnostic tool for evaluation of ventricular volumes, function, and extent of myocardial fibrosis in this population.^2^ In patients with ICDs, however, CMR is underutilized because of concern for device malfunction caused by radiofrequency energy deposited during image acquisition. The pulsed radiofrequency energy can cause vibration, heating, and/or dislocation of electrodes; prevent capacitors from delivering shocks; shorten battery life; or even induce malignant arrhythmias.^3^ More recently, “magnetic resonance imaging (MRI)–conditional” devices were developed to allow continued function during CMR image acquisition.

**Figure 1:**
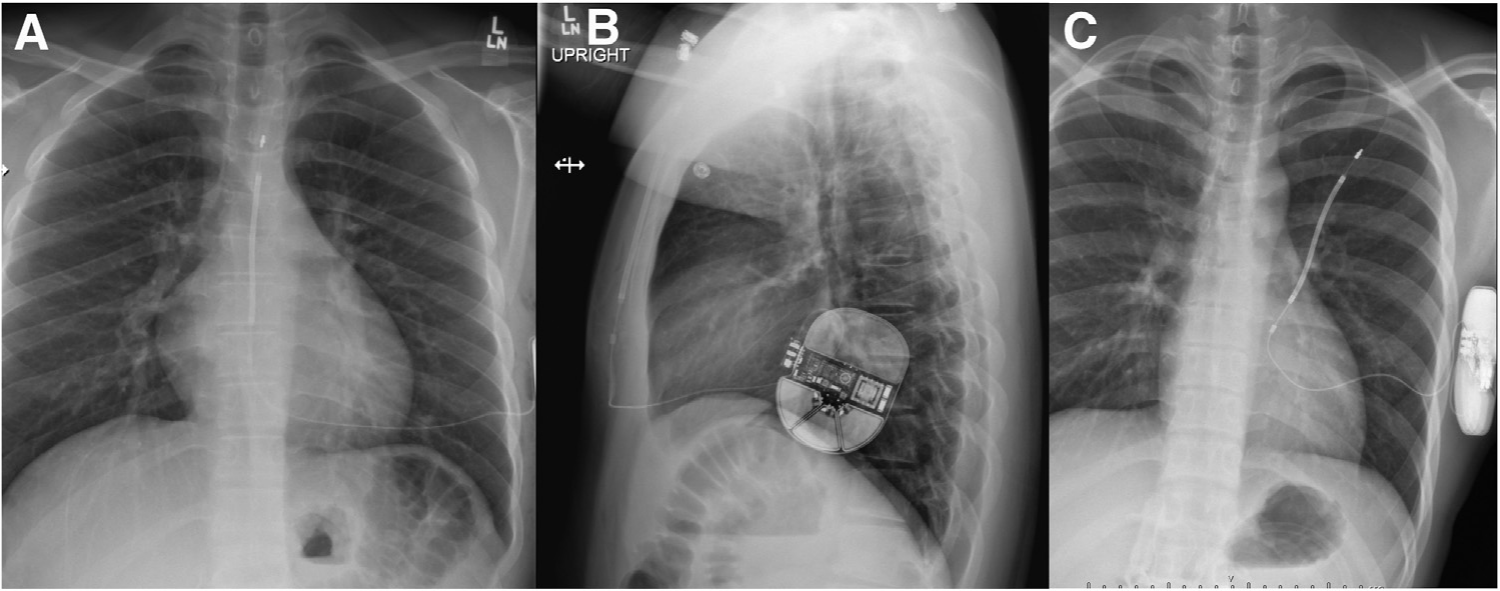
Anteroposterior and lateral chest radiographs. **A:** The conductor coil of the subcutaneous implantable cardioverter-defibrillator (SICD) is subcutaneously tunneled from the anterior chest wall to the xiphoid process. **B:** The conductor is then subcutaneously tunneled to the left axilla, where the generator is placed. **C:** This patient did not achieve appropriate SICD sensing and defibrillation on initial device placement because the coil and generator did not vector across the majority of ventricular myocardium.

The relatively newer subcutaneous ICD (SICD) was brought to market to provide equivalent therapy to conventional ICDs for life-threatening arrhythmias, while mitigating the potential complications of transvenous ICDs. These complications include endovascular infection, venous thrombosis, lead failure, conductor fracture leading to inappropriate shocks, and the inherent risks of lead extraction.^4^ The SICD is implanted by tunneling the conductor coil subcutaneously into the anterior chest wall superiorly from the xiphoid process (Figure 1A). The conductor is then tunneled subcutaneously to a generator in the left axillary position (Figure 1B). The sensing of arrhythmias is accomplished in a similar fashion to a surface electrocardiogram by creating bipolar electrograms from 2 electrodes on the lead to the generator, or between the 2 electrodes. Therapy is provided by delivering a high-voltage shock from the coil on the anterior chest wall to the generator.^5^ The SICD has been championed by the pediatric electrophysiology community and lead extractionists.^6^

Following the industry trend for transvenous ICDs, device manufacturers developed and tested the SICD to achieve MRI conditionality for all types of MRI, including CMR. However, MRI conditionality is a safety designation only, and does not address actual image quality or other performance measures of CMR imaging. This case series of pediatric patients with subcutaneous ICDs who underwent CMR describes the effects of the SICD on image quality while documenting the safety of the procedure.

## Case report

The 4 patients presented were 14–17 years old (mean and median 16 years), weighed 53–108 kg (mean 75.2 kg; median 80 kg), and had a body surface area of 1.54–2.31 m^2^ (mean 1.89 m^2^; median 1.99 m^2^). Patients underwent clinically indicated CMR an average of 447 days (range 52–811 days) after SICD implantation. There were 5 total CMR studies performed on the 4 patients in the cohort. These patients included 2 with arrhythmogenic cardiomyopathy, 1 with hypertrophic cardiomyopathy, and 1 with idiopathic dilated cardiomyopathy. A Siemens 1.5 T Espree scanner (Siemens Medical Solutions Inc, Malvern, PA) was used along with standard cine steady-state free precession (SSFP) sequences in multiple planes. Gradient echo (GRE) cine sequences were also attempted when SSFP cine sequences were significantly degraded by metallic artifact. The maximum specific absorption rate for the studies was maintained below 2 W/kg. The studies were directly supervised by an attending cardiologist and a device representative capable of interrogating and reprogramming the device. In selected studies, an intravenous (IV) gadolinium-based contrast agent (Magnevist; Bayer HealthCare Pharmaceuticals Inc, Wayne, NJ) was injected for assessment of late gadolinium enhancement. Measurements of ventricular volumes, mass, and function were performed by an experienced MRI cardiologist (PR) using standardized techniques and commercially available software (CMR 42; Circle Cardiovascular Imaging, Calgary, Canada). This case series was granted exemption from review by the CHOC Children’s Hospital Institutional Review Board.

The Emblem A219 CMR SICD (Boston Scientific Corporation, Marlborough, MA) device was implanted in 3 patients. The other received the SQ-RX Model 1010 (Cameron Health Inc, San Clemente, CA). We used Boston Scientific 3501, Boston Scientific 3010, or Cameron Health Q-TRAK 3400 leads. SICD lead placement was verified under fluoroscopy. No device complications occurred between implantation and CMR.

**Figure 2:**
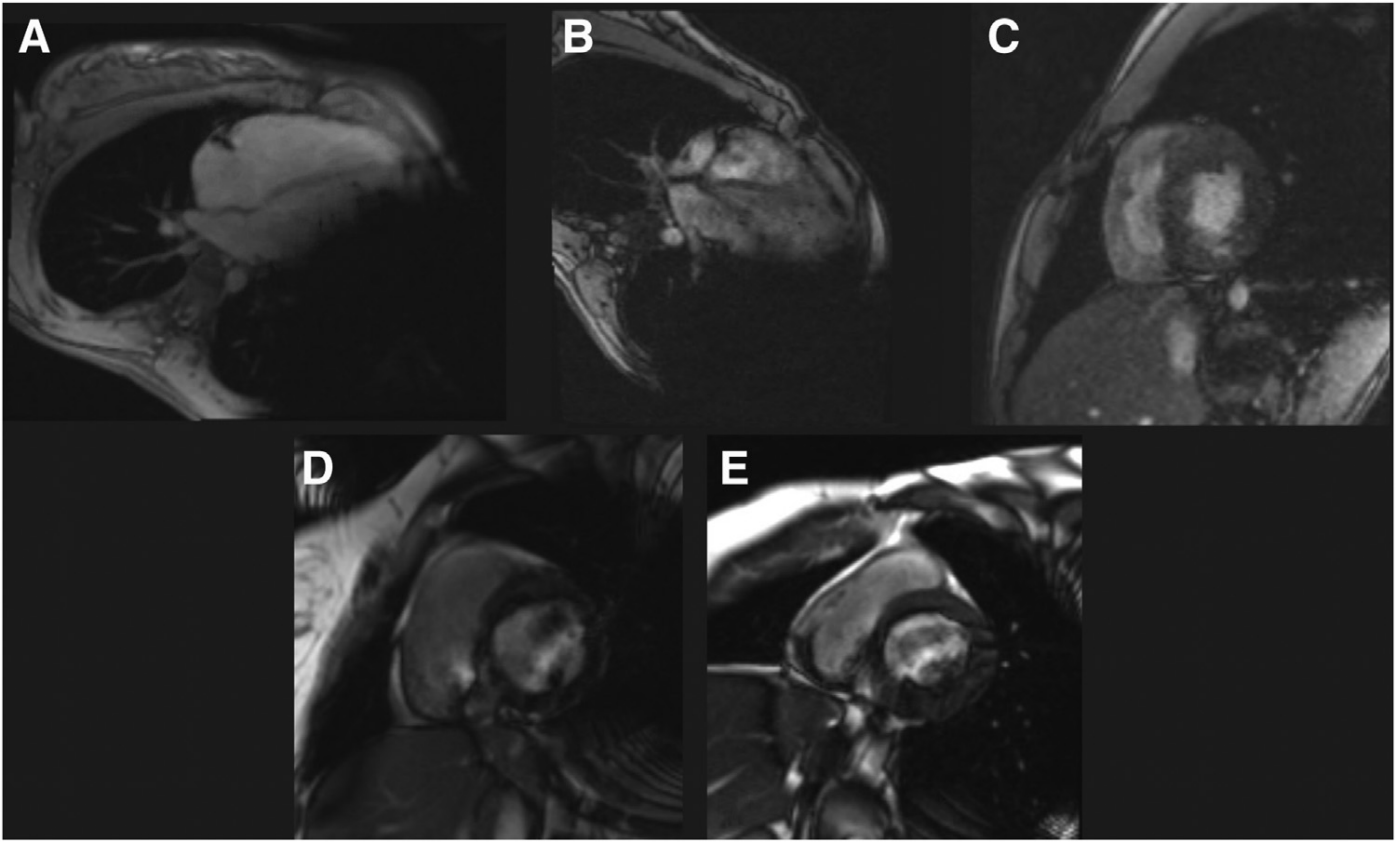
Referred sample cardiac magnetic resonance images for each patient from Table 1.

**Figure 3:**
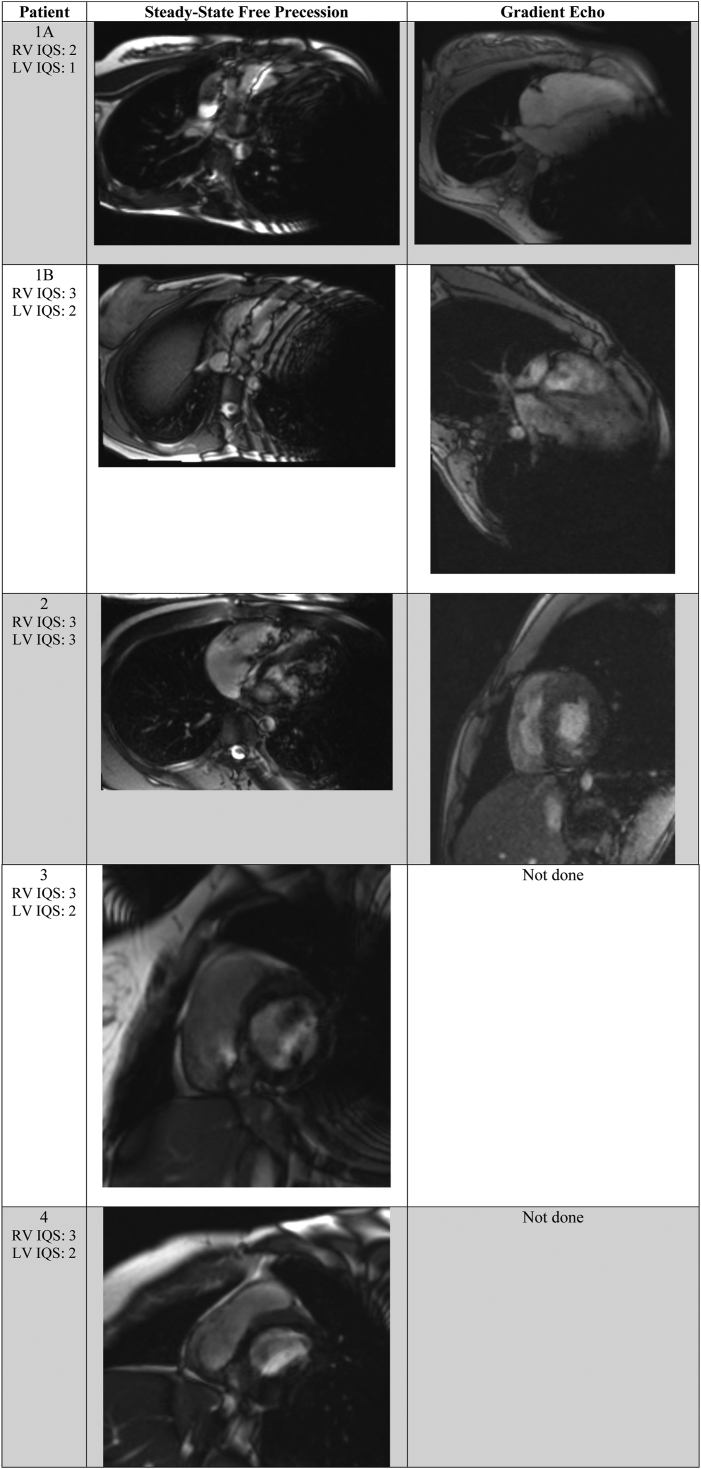
Image quality score (IQS) shown alongside corresponding cardiac magnetic resonance images, including steady-state free precession sequences and gradient echo sequences. LV = left ventricle; RV = right ventricle.

The SICD was interrogated prior to and following completion of the CMR per laboratory protocol. All SICDs were placed on “MRI mode” during image acquisition, which inhibits sensing and therapy delivery. Afterwards, all devices were programmed to the pre-CMR settings. The images were reviewed and postprocessed by the cardiologist. Each CMR was then assigned an Image Quality Score (IQS) based on ability to quantitatively evaluate left and right ventricular volumes, function, and mass. The IQS was adapted from the method employed by Han and colleagues.^7^ IQS in our study ranged from “nondiagnostic” (1), with poor visualization of ventricular chambers, to “good quality” (4), with clearly identified chambers and distinct trabeculae and endocardium (Table 1, Figure 2). The 5 studies in this cohort, including the IQS and obtainable diagnostic information, are detailed in Figure 3.

**Table 1:** Patient demographics, clinical results, and image quality score of each cardiac magnetic resonance imaging study

Patient	BSA (m^2^)	Days between SICD implant & CMR	Indications for CMR	Clinical question answered? (Y/N)	RV qualitative (size + function)	RV quantitative	RV IQS†	LV qualitative (size + function)	LV quantitative	LV IQS†	Sample image
1A	1.54	382	CMR 1: Evaluate ARVD	No	Yes	No	2	No	No	1	Figure 2A
1B	1.56	811	CMR 2: Evaluate ARVD		Yes	Yes	3	Yes	No	2	Figure 2B
2	2.04	442	Hypertrophic cardiomyopathy	Yes	Yes	Yes	3	Yes	Yes	3	Figure 2C
3	1.99	52	Evaluate ventricular function in setting of recurrent myocarditis	No; could not evaluate for LV enlargement	Yes	Yes	3	Yes	No	2	Figure 2D
4	2.31	547	Evaluate ARVD & subendocardial ischemia	No; RV free wall visualized but no reproducible volume could be obtained	Yes	Yes	3	No	No	2	Figure 2E
Mean	1.89	446.8					2.8			2	
Median	1.99	442					3			2	

ARVC = arrhythmogenic right ventricular cardiomyopathy; BSA = body surface area; CMR = cardiac magnetic resonance imaging; IQS = image quality score; LV = left ventricle; RV = right ventricle; SICD = subcutaneous implantable cardioverter-defibrillator.

† IQS: 1 = nondiagnostic (poor visualization of ventricular chambers); 2 = poor quality (chamber walls seen but poorly defined); 3 = moderate quality (chambers clearly identified but with suboptimal definition of the endocardium and trabeculae); 4 = good quality (chambers clearly identified with good definition of the endocardium and trabeculae).

There were no adverse outcomes or unintended changes to device settings, battery life, or lead impedance immediately post CMR. One patient did have a 14% decrease in battery life over the following 9 months without any ICD discharges or dysrhythmias in that time. Two patients experienced inappropriate discharges 4 months and 6 months after their CMR but had no change in SICD lead or generator function after the CMR compared to immediately prior to the CMR; they eventually underwent device replacement. Patient 4 reported a brief sensation of heating during CMR in the area of the device, which quickly resolved with shortening of the image acquisition time.

All 4 patients had CMR image quality adversely affected by susceptibility artifacts from the SICD and/or lead (Table 1, Figure 2). The IQS for these patients were between 1 and 3 (mean 2; median 2) for left ventricular analysis and 2 and 3 (mean 2.8; median 3) for the right ventricle (Table 1).

Quantitative left ventricular volumes, function, and mass could only be successfully calculated in 1 case (20%). In 3 of the 5 cases (60%), a limited assessment of qualitative function was reported. However, in 2 of the patients, imaging artifact was significant enough to completely preclude assessment of the left ventricle. Left ventricular quantitative assessment was impeded by generator-induced artifact in both the short and long axis imaging planes, predominantly in the region of the midventricle and apex. The ventricular slices more distal to the generator—namely, the basal segments—retained some definition but remained insufficient to answer clinical questions that would direct patient management. For the right ventricle, however, standard quantitative assessment was possible in all patients, and in all but 1 study (80%). In that study, limited *qualitative* assessment of right ventricular size and systolic function was still possible. In 3 of the studies, IV contrast was not given owing to significant image degradation. In the 2 other studies, IV contrast was given, but the sequences were ultimately noninterpretable. Ultimately, the clinical question could only be answered by CMR in 25% of the patients and in only 20% of the studies in our cohort.

## Discussion

Our findings suggest that although performing CMR in patients with MRI-conditional SICDs using carefully designed protocols is safe, significant and clinically relevant distortion of the images can occur. Left ventricular IQS was of poor quality for all but 1 study. However, right ventricular IQS was of moderate quality for all but 1 study. This may be beneficial in patients with classical phenotype arrhythmogenic cardiomyopathy, where diagnosis and follow-up are guided by specific criteria based on primarily quantitative CMR right ventricular parameters. In the case of left-dominant or biventricular phenotypes of arrhythmogenic cardiomyopathy, however, our study suggests CMR would have limited clinical benefit. In conditions primarily involving the left ventricle, such as hypertrophic or dilated cardiomyopathy, we found CMR in the presence of an SICD of very limited utility.

Significant loss of useful data occurred in 80% of the studies, leading to the clinical questions not being answered. We obtained at least qualitative size and function information of the right ventricle in all cases, and obtained limited quantitative information of the right ventricle in 80% of the cases. Therefore, owing to suboptimal CMR image quality occurring after SICD implantation, we would recommend that CMR be obtained prior to device implantation, if clinically indicated.

The administration of IV contrast agent and subsequent myocardial delayed gadolinium enhancement imaging was not attempted for 3 of 5 CMR studies in our series due to severe image distortion. In the remaining 2 studies with delayed enhancement imaging, image distortion was too significant to yield meaningful information. We would thus recommend forgoing contrast administration unless scout images are first carefully reviewed for image degradation.

The use of postcontrast GRE cine sequences can ameliorate the image-degrading effects of metallic artifacts and thus offers a reasonable assessment of myocardial volumes and function when SSFP cine images are nondiagnostic.^8^ We had some success in visualizing the left ventricular myocardium in our patients using GRE when SSFP image quality was poor (sequence parameters are included in Appendix). In terms of myocardial fibrosis imaging, modified sequences have been developed that may improve visualization of delayed gadolinium enhancement. For example, Shao and colleagues^9^ have shown that wideband FLASH-MOLLI sequences may be reasonable alternatives to standard phase-sensitive inversion recovery for better myocardial visualization in the presence of metallic artifacts. Currently, such sequences are not widely available.

SICD positioning in the body, and its positioning in relation to the anatomic area of interest during CMR, may play a role in the extent of the area of image distortion. The device is typically positioned with sensing electrodes and a high-voltage coil in the subcutaneous anterior chest and the generator in the left axilla. This configuration is crucial for appropriate sensing and effective defibrillation, which requires the passage of direct current from the coil through the majority of ventricular myocardium to the generator. Unfortunately, this configuration also aligns the resulting image artifact over the ventricular mass, leading to image distortion in locations that are critical for accurate CMR data analysis (Figure 1C).

Based on analysis of this case series, we hypothesize that the proximity of the SICD generator to the apex of the heart, combined with the larger size of the SICD generator (59.5 cm^2^) compared to MRI-conditional transvenous single-lead ICDs (26.5–35 cm^2^) and the smaller body surface area of younger patients, may all contribute to CMR image artifacts, particularly those affecting the more posterolaterally positioned left ventricle. It is likely that CMRs done on patients with other types of cardiovascular implantable electronic devices may also be negatively affected in a similar fashion as SICDs.

## Conclusion

We present the first pediatric case series of patients with MRI-conditional SICDs who underwent CMR at 1.5 T with a focus on the adverse effect of the device on image quality and diminution of the diagnostic value of the resultant images. Accordingly, we were unable to derive consistent quantitative ventricular data and myocardial fibrosis assessment by CMR in our cohort. Alternative sequences such as postcontrast GRE can provide qualitative information in some patients. Additionally, significant imaging artifact was more present over the left ventricle. The right ventricle was better visualized, leading to the conclusion that CMR may continue to be useful in SICD patients in whom primarily right ventricular pathology is present.
