# Supplementary material for: Influence of subcutaneous implantable defibrillators on cardiovascular magnetic resonance image quality in pediatric patients
Source: HeartRhythm Case Rep. 2022 Apr 26;8(7):509–14. doi: 10.1016/j.hrcr.2022.04.014 (PMC9289066; doi:10.1016/j.hrcr.2022.04.014)
Supplement: Appendix [file mmc1.docx]

**Appendix – Cardiac MRI sequence parameters**

Steady-state free precession (SSFP):

- TR = 26.3 ms
- TE = 1.22 ms
- Flip angle 72°
- Bandwidth = 1002 Hz/pixel
- FOV = 320 x 96 mm, spatial resolution = 2.2 × 1.7 mm, slice thickness = 5 mm with no interslice gap
- 30 phases per cardiac cycle
- Acceleration factor = 2

Gradient-echo (GRE):

- TR = 61.6 ms
- TE = 2.64 ms
- Flip angle 15°
- Bandwidth = 501 Hz/pixel
- FOV = 300 x 78 mm, spatial resolution = 2.2 × 1.6 mm, slice thickness = 8 mm with no interslice gap
- 30 phases per cardiac cycle
- Acceleration factor = 2
